# Glutaminase Inhibition on NSCLC Depends on Extracellular Alanine Exploitation

**DOI:** 10.3390/cells9081766

**Published:** 2020-07-23

**Authors:** Elisa Caiola, Marika Colombo, Giovanna Sestito, Monica Lupi, Mirko Marabese, Roberta Pastorelli, Massimo Broggini, Laura Brunelli

**Affiliations:** 1 Laboratory of Molecular Pharmacology, Department of Oncology, Istituto di Ricerche Farmacologiche Mario Negri IRCCS, 20156 Milan, Italy; elisa.caiola@marionegri.it (E.C.); Marika.colombo@marionegri.it (M.C.); massimo.broggini@marionegri.it (M.B.); 2 Laboratory of Mass Spectrometry, Department of Environmental Health Sciences, Istituto di Ricerche Farmacologiche Mario Negri IRCCS, 20156 Milan, Italy; giovanna.sestito@marionegri.it (G.S.); roberta.pastorelli@marionegri.it (R.P.); 3 Laboratory of Antitumor Pharmacology, Department of Oncology, Istituto di Ricerche Farmacologiche Mario Negri IRCCS, 20156 Milan, Italy; monica.lupi@marionegri.it

**Keywords:** NSCLC, glutaminase inhibition, metabolism, alanine uptake, cancer cell resistance, alanine aminotransferase

## Abstract

Non-small-cell lung cancer (NSCLC) cell lines vary in their sensitivity to glutaminase inhibitors, so it is important to identify the metabolic assets underling their efficacy in cancer cells. Even though specific genetic lesions such as in KRAS and LKB1 have been associated with reliance on glutamine for their metabolic needs, we found no distinction between glutaminase inhibitor CB-839 sensitivity and resistant phenotypes in NSCLC cells with or without these genetic alterations. We demonstrated the close relationship between environmental alanine uptake and catabolism. This response depended on the individual cell’s ability to employ alanine aminotransferase (GPT2) to compensate the reduced glutamate availability. It may, therefore, be useful to determine GPT2 levels to predict which NSCLC patients would benefit most from glutaminase inhibitor treatment.

## 1. Introduction

Non-small-cell lung cancer (NSCLC) cells rely on glutamine metabolism to support cancer metabolism and energy production [1,2,3]. Glutamine and its metabolic conversion to glutamate contribute to the generation of mitochondrial ATP, lipid biosynthesis and the redox balance for cell survival and proliferation [4]. The importance of these metabolic branches makes glutamine metabolism an attractive target for therapeutic strategies. Several selective inhibitors of glutaminase (GLS), the rate-limiting enzyme responsible for the deamination of glutamine to glutamate, such as BPTES or CB-839, have now been tested in several pre-clinical cancer models including NSCLC, breast, pancreatic, renal, leukemia and lymphoma [5]. BPTES and CB-839 both lower the level of glutamate and their downstream mitochondrial metabolites such as tricarboxylic acid cycle (TCA) cycle intermediates, aspartate and GSH, inducing an anti-tumor effect in both in vitro and in vivo preclinical models [6]. The selective GLS1 inhibitor CB-839 is currently undergoing clinical trials in several human malignancies, including NSCLC. Despite the in vitro effectiveness of the CB-839 inhibitor in counteracting NSCLC cell proliferation, different NSCLC cell lines vary in their responses [7]. Therefore, it is important to identify the intracellular metabolic axis responsible for the glutaminase sensitivity or resistance to predict the response to glutaminase inhibition and tailor effective anticancer treatments.

We investigated the metabolic response to CB-839 in a panel of NSCLC cell lines and examined how specific metabolic traits contributed to the activity of CB-839. The findings confirm that NSCLC cells diverge towards CB-839 either sensitive or resistant phenotypes, with no distinction between NSCLC cells with KRAS mutations or LKB1 loss either in single or concomitant genetic lesions. Environmental alanine uptake and metabolism therefore appeared to be the metabolic route through which some NSCLC cell lines overcome CB-839 inhibition. We also found that inhibiting alanine catabolism negatively affected NSCLC CB-839 resistant cell growth and proliferation.

## 2. Methods

### 2.1. Cell Culture and Treatment

NSCLC cell lines (H1792, H1975, H358, H2030, H520, H23, H1650, H460, A549, LU99) were grown in in Roswell Park Memorial Institute (RPMI-1640) medium supplemented with 10% fetal bovine serum (FBS). H1299-derived clones (KRASwt/LKB1wt, KRASmut/LKB1wt, KRASwt/LKB1del and KRASmut/LKB1del) were grown in RPMI-1640 10% FBS medium with 500 µg/mL of G418 (Thermo Fisher Scientific, Waltham, MA, USA). LU99-2 and H358-7 LKB1 KO clones were generated using LKB1 CRISPR/Cas9 KO plasmid and HDR plasmid (Santa Cruz Biotechnology, Dallas, TX, USA). Cell lines and clones were routinely tested by PCR for mycoplasma contamination. They were also authenticated with the PowerPlex 16 HS System (Promega, Madison, WI, USA) every six months by comparing the Short Tandem Repeats (STR) profiles with those deposited in the American Type Culture Collection (ATCC) and/or Deutsche Sammlung von Mikroorganismen und Zellkulturen (DSMZ, German Collection of Microorganisms and Cell Cultures) databases. CB-839 (TargetMol, Wellesley Hills, MA, USA) DMSO stock solutions (10 mM) were dissolved in medium just before use. l-cycloserine (Santa Cruz Biotechnology, Dallas, TX, USA) was dissolved in sterile water and diluted in medium just before use. RPMI-1640 with pyruvate (Microgem, Napoli, Italy) was added to cells in culture about one week before the cytotoxicity experiments. In all the cytotoxicity experiments, with either single or combined treatments, cells were continuously treated for 72 h. Drug effects were assessed by the CellTiter Aqueous 3-(4,5-dimethylthiazol-2-yl)-5-(3-carboxymethoxyphenyl)-2-(4-sulfophenyl)-2H-tetrazolium (MTS) assay as previously reported [8] and/or by the sulforhodamine B (SRB) assay following the manufacturer’s instructions. Briefly, cells were fixed in 10% trichloroacetic acid. After 24 h, plates were colored with 0.4% SRB, washed with 1% acetic acid and left thawing at room temperature. Then, the dye was solubilized with 10 mM Tris base, and the absorbance was read at 560 nM with a Glomax Discover microplate reader (Promega, Madison, WI, USA). The mean and SD of at least three independent experiments, each consisting of six replicates, were presented.

### 2.2. Flow Cytometric Analysis of DNA Content

Cells were seeded at the desired concentrations and after 24 h were treated with CB-839 500 nM for 24 or 48 h. About 1 × 10^6^ cells were fixed in ice-cold 70% ethanol, washed in PBS, resuspended in 1 mL of 25 μg/mL of propidium iodide (Merck Millipore, Burlington, MA, USA) and 12.5 μL of RNase (1 mg/mL) (Merck Millipore, Burlington, MA, USA) and stained for 2 h at room temperature in the dark. Cell cycle analysis was performed on at least 10,000 cells for each sample using the FACS Calibur (Becton Dickinson, Franklin Lakes, NJ, USA).

### 2.3. Metabolomics Analysis: 13C Labelling

As previously described in [9], 13C_5_-labelled-glutamine analysis was carried out on NSCLC H1299 derived cell clones. Briefly, NSCLC cell clones (1 × 10^6^ cells) were cultured for 24 h in RPMI -1640 media supplemented with 10% dialyzed FBS and 1% standard glutamine; then, cells were treated with CB-839 (500 nM), and after 18 h, medium was replenished with RPMI with 2 mM 13C_5_-glutamine (Cambridge Isotopes Laboratories, Tewksbury, MA, USA) and CB-839 (500 nM) for 6 h. To trace alanine metabolism, NSCLC H1299-R1 and -R2 derived cell clones, and LU99, A549, H358 and H520 cell lines were seeded at the desired concentrations and, after 24 h, medium was supplemented with 1 mM U-13C_3_-alanine and CB-839 (500 nM) for 24 h. After labelling and treatment, conditioned culture media were collected, cells were rinsed, and metabolism was quenched with liquid nitrogen. Metabolites were extracted using MeOH:ACN:water (50:30:20), and 8 μL of supernatant was collected for liquid chromatography tandem mass spectrometry (LC–MS/MS, LTQ-Orbitrap XL, Thermo Fisher Scientific, Waltham, MA, USA). An Atlantis T3 column (3.5 μm, 150 × 2.1 mm, Waters, Milford, MA, USA) was used for LC separation, and the detection of metabolites was performed using a LTQ-Orbitrap XL mass spectrometer with electrospray (ESI) ionization, examining metabolites in both positive and negative ion modes over the mass range of 75–1000 *m*/*z*. The mobile phase for elution was a gradient established between water acidified with 0.1% formic acid (positive), 10 mM ammonium formiate (negative) (A) and acetonitrile (B) at a flow rate of 150 μL/min. Retention times of all metabolites of interest were validated using pure standards. The exact masses and all isotopologue combinations (M + 0, M + 1, M + 2, M + 3, M + 4, M + 5) of the metabolites of interest were extracted from full-scan data, with a 2 ppm window, and peak areas were integrated using TraceFinder 3.1 (Thermo Fisher Scientific, Waltham, MA, USA). The measured distribution of mass isotopomers was corrected for natural abundance of 13C and normalized against the cell numbers. For 13C_5_-glutamine-derived labelling, unlabeled M + 0 and M + 2, M + 3, M + 4, M + 5 labelled glutamine, glutamate, succinate, fumarate, malate, citrate and aspartate were monitored. For 13C_3_-alanine-derived labelling, unlabeled M + 0 and M + 2, M + 3, M + 4, M + 5 labelled alanine, malate, citrate, glutamate and glutathione were monitored.

### 2.4. Targeted Metabolite Measurement in Culture Conditioned Media

Conditioned culture media from NSCLC cells (H1792, H1975, H358, H2030, H520, H23, H1650, H460, A549, LU99), either CB-839 treated or untreated, were collected. Proteins were precipitated with cold MeOH and centrifuged, and 8 µL of supernatant were examined by liquid chromatography tandem mass spectrometry targeted metabolomics to determine glucose, alanine, lactate, glutamate and aspartate levels. An Atlantis T3 column (3.5 μm, 150 × 2.1 mm, Waters, Milford, MA, USA) was used for LC separation and the detection of these metabolites by using an LTQ-Orbitrap XL mass spectrometer (Thermo Fisher Scientific, Waltham, MA, USA) with electrospray (ESI) ionization, examining metabolites in both positive (alanine, aspartate, glutamate) and negative (glucose and lactate) ion modes, over the mass range of 75–1000 *m*/*z*. The mobile phase for elution was a gradient established between water acidified with 0.1% formic acid (positive), 10 mM ammonium formiate (negative) (A) and acetonitrile (B) at a flow rate of 150 μL/min. Retention times of all metabolites of interest were validated using pure standards. The exact masses of the metabolites of interest were extracted from full-scan data, with a 2 ppm window, and peak areas were manually integrated using TraceFinder 3.1 (Thermo Fisher Scientific, Waltham, MA, USA) and normalized against the cell numbers.

### 2.5. Free Fatty Acids (FFA)

FFAs (palmitic, oleic and stearic) were measured in untreated and CB-839-treated NSCLC H1299-derived cell clones (1 × 10^6^ cells). FFAs were extracted with 50 μL of cold MeOH, and 10 μL of internal standard (13C-palmitic acid 10 ng, Merck Millipore, Burlington, MA, USA) was added. Samples were vortexed for 30 s and incubated for 10 min in ice. After centrifugation (15,000× *g*, 15 min at 4 °C), the supernatant was collected in glass vials and analyzed by liquid chromatography tandem mass spectrometry (LC–MS/MS, LTQ-Orbitrap XL, Thermo Fisher Scientific, Waltham, MA, USA). HPLC separation was done on an Ascentis Express C18 column, 150 × 2.1 mm, 2.7 μm particle size (Merck Millipore, Burlington, MA, USA), maintained at 37 °C. A dual eluent system consisting of ammonium acetate 5 mM in ultrapure water (A) and ammonium acetate 5 mM in methanol (B) was pumped at a flow rate of 150 µL/min. The elution gradient was as follows: 0 min (80% B), 20 min (88% B), 28 min (100% B), 38 min (100% B), 39 min (80% B). The injection volume was 8 µL. The exact masses of FFA were extracted from full-scan data with a 5 ppm window, and peak areas were manually integrated using an Xcalibur Browser (Thermo Fisher Scientific, Waltham, MA, USA) and normalized against 13C-palmitic acid, assuming that all FFA had the same extraction recovery and instrumental response and cell numbers.

### 2.6. Western Blotting Analyses

Cell pellets were lysed in the lysis buffer [10] added with protease and phosphatase inhibitor cocktail for 30 min on ice. Insoluble material was pelleted at 10,000× *g* for 15 min at 4 °C, and the protein concentration was determined using a BioRad assay kit (BioRad, Hercules, CA, USA). Thirty μg of total cellular proteins were separated on SDS-PAGE and electrotransferred to activated PVDF membrane (Merck Millipore, Burlington, MA, USA). Immuno-blotting was carried out with anti-GLS1 (Cell signaling, 1:1000), anti-GPT2 (Santa Cruz Biotechnology, Dallas, TX, USA 1:250) and anti-RAN (Santa Cruz Biotechnology, Dallas, TX, USA 1:500) primary antibodies and anti-mouse and anti-rabbit peroxidase labelled secondary antibodies (BioRad, Hercules, CA, USA). Horseradish-peroxidase substrate (ECL Western Blotting Detection, Amersham-Life Science, Little Chalfont, UK) was added and the signal was revealed through an Odyssey Fc instrument (LI-COR, Lincoln, NE, USA). 

### 2.7. Statistical Analysis

All statistical analyses were done using Prism (V8, GraphPad, San Diego, CA, USA). We used the non-parametric Wilcoxon Mann–Whitney test when comparing two groups and one or two-way ANOVA and Bonferroni post-test when comparing three or more. 

## 3. Results and Discussion

### 3.1. The Heterogeneous Response to Glutaminase Inhibition of NSCLC Cell Lines Does Not Depend on Genetic Backgrounds or Glycolytic Rebound

**Figure 1:**
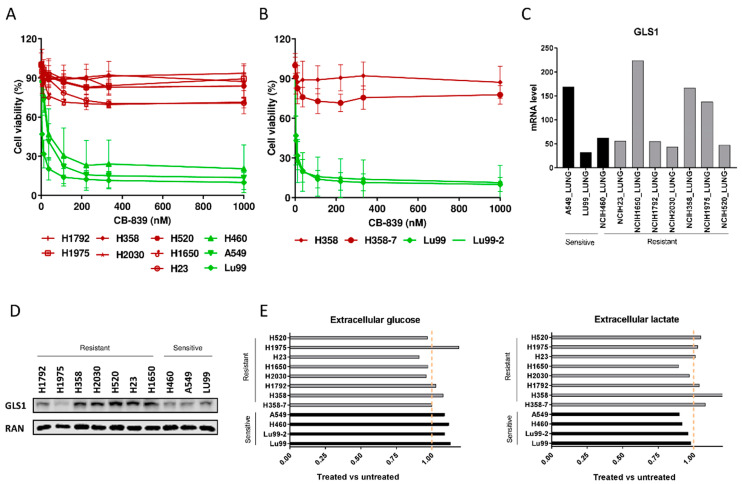
(**A**) Dose–response curves of the NSCLC cell lines panel treated with increasing concentrations of CB-839. The response to the drug was assessed 72 h from the start of treatment with the MTS assay. The average of three independent experiments is reported. (**B**) Dose–response curves of the NSCLC LU99 and H358 LKB1 isogenic systems treated with increasing concentrations of CB-839. The response to the drug was assessed 72 h from the start of treatment with the MTS assay. The average of three independent experiments is reported. (**C**) GLS1 RNAseq gene expression data retrieved from the CCLE [17], in ten NSCLC cell lines. (**D**) Western Blot analysis of GLS1 protein levels in the ten NSCLC cell lines used. Ran was used as loading control. The figure is representative of at least three independent experiments. (**E**) Fold change in abundance (normalized peak area) of extracellular glucose uptake and lactate release in NSCLC CB-839 treated vs. untreated cells (500 nM CB-839, 6 h treatment). Mean ± SD of triplicate culture/conditions.

To extend the therapeutic utility of glutamine dependence in NSCLC, we impaired GLS activity by using CB-839, a GLS1 inhibitor [11]. Since the influence of *KRAS* and *LKB1* alterations on metabolic reprogramming and tumor growth [7,9,12,13,14] has already been documented, we selected a panel of ten NSCLC cell lines with different combinations of these genetic alterations (Table 1). In line with recent data [7,13], our NSCLC cell lines varied in their sensitivity to the CB-839 glutaminase inhibitor, even when cells were analyzed using both metabolism-dependent and -independent cell viability assays (Figure 1A and Supplementary Figure S1A). However, in our cell lines, the CB-839 response seemed unrelated to the single or concomitant presence of *KRAS* mutations and copy number variations or LKB1 loss of function. To further elucidate the mechanism leading to the clear antiproliferative effect observed in sensitive cell lines treated with CB-839, flow cytometric analysis of DNA content was performed. DNA histograms of control and treated samples were very similar in both sensitive and resistant cell lines; hence, we supposed that CB-839 induced a generalized delay in all cell cycle phases in sensitive cells (Supplementary Figure S1B).

Moreover, to clarify the involvement of single or concomitant *KRAS* mutation and LKB1 loss in triggering NSCLC cell response to GLS1 inhibition in a homogeneous genetic background, we treated with CB-839 NSCLC isogenic clones, LU99-2 and H358-7, generated from LU99 and H358 cell lines, respectively, with *LKB1* deletion obtained by the CRISPR/Cas9 technique [15]. The treatment response of LU99- and H358-derived isogenic systems seemed independent from the LKB1 alterations. In fact, the parental cell lines and their clones behaved similarly after GLS1 pharmacological inhibition, independently from the presence or absence of LKB1. LU99 and LU99-2 cell lines were the most sensitive to the treatment, and the deletion of *LKB1* in H358 cells (not responding to CB-839) did not sensitize them to the drug (Figure 1B). Galan-Cobo et al. [7] and Romero et al [13] reported that almost all their KRAS/LKB1/KEAP1 (KLK) altered cell lines were sensitive to GLS1 inhibition. Even considering the *KEAP1* status in our NSCLC cell lines (Table 1), we did not see any enrichment of KLK mutated cell lines in the responding group (Figure 1A). However, we used a restricted panel of cells and stringent criteria (about 50% reduction of cell viability) to distinguish sensitive from resistant cells. 

We also checked whether the different response to CB-839 treatment in our NSCLC cells was due to a different basal expression of GLS1. The expression data from the Cancer Cell Line Encyclopedia (CCLE) and from our Western blot analyses excluded this, considering that both the sensitive and the resistant cell lines showed comparably heterogeneous GLS1 mRNA and protein levels (Figure 1C,D). We also excluded the possibility that resistant cells could increase the glycolytic axis to overcome the glutamine dependence; all the NSCLC-treated cells had comparable uptake and release of glucose and lactate in the extracellular compartment, independently from their response to treatment (Figure 1E). 

### 3.2. The GLS1-Resistant Cells Rely on Extracellular Alanine to Sustain Their Viability

**Figure 2:**
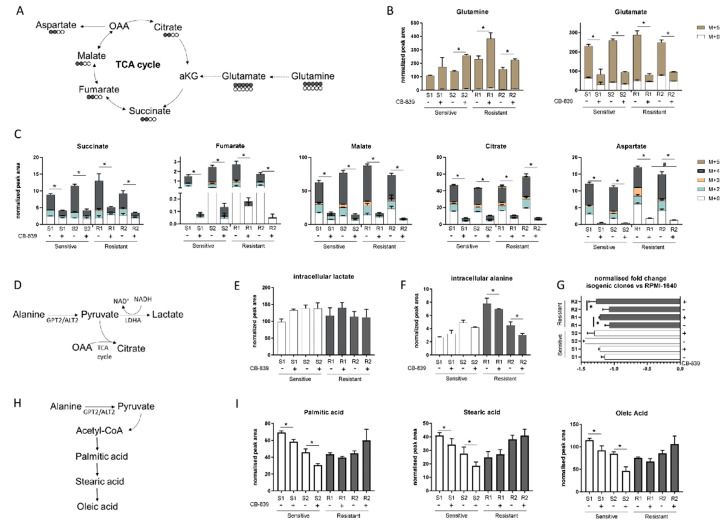
(**A**) Schematic representation of the 13-C glutamine labelling experiment. (**B**) M + 0 and M + 5 glutamine and glutamate abundance in two sensitive and two resistant H1299-derived clones with or without CB-839 (500 nM, 24 h treatment). (**C**) Isotope labelling of the TCA cycle in the presence of 13C_5_-glutamine with or without CB-839 (500 nM, 24 h treatment) in two sensitive (S1, KRASwt/LKB1wt, S2, KRASmut/LKB1wt) and two resistant (R1, KRASwt/LKB1del, R2, KRASmut/LKB1del) H1299-derived clones (normalized peak area). (**D**) Schematic representation of intracellular pyruvate metabolism. (**E**) Intracellular lactate levels (normalized peak area) with or without CB-839 (500 nM, 24 h treatment) in two sensitive and two resistant H1299-derived clones. (**F**) Intracellular alanine levels (normalized peak area) with or without CB-839 (500 nM, 24 h treatment) in two sensitive and two resistant H1299-derived clones. (**G**) Normalized fold change of extracellular alanine levels vs. unconditioned RPMI-1640 with or without CB-839 (500 nM, 24 h treatment) in two sensitive and two resistant H1299-derived clones. (**H**) Schematic representation of fatty acid biosynthesis. (**I**) Intracellular levels of fatty acids with or without CB-839 (500 nM, 24 h treatment) in two sensitive and two resistant H1299-derived clones. Mean ± SD of triplicate culture/conditions. Statistical significance * *p* < 0.05 untreated vs CB-839 treated cells, ^#^ *p* < 0.05 untreated sensitive vs untreated resistant cells, Wilcoxon Mann–Whitney test (M + 0, M + 2, M + 3, M + 4, M + 5).

Given the lack of correlation between specific genetic alterations and CB-839 sensitivity, we wondered whether different intracellular metabolic configurations in the sensitive and resistant cell lines could be responsible for the different behavior after GLS1 treatment. We used our already metabolically profiled NSCLC H1299 isogenic cell clones [9] to test the CB-839 inhibition efficacy. For the H1299 cell line, we had four different genotypes (KRASwt/LKB1wt, KRASmut/LKB1wt, KRASwt/LKB1del and KRASmut/LKB1del), so we could show different responses to CB-839, dividing them into two sensitive and two resistant phenotypes, according to *LKB1* status and independently from the presence of *KRAS* alterations (Supplementary Figure S2). To assess the intracellular metabolic axis responsible for the glutaminase sensitivity or resistance, we traced the 13C_5_-glutamine (Gln) (substrate of GLS1) intracellular fate (Figure 2A) in the two resistant (R1: KRASwt/LKB1del, R2: KRASmut/LKB1del) and two sensitive (S1: KRASwt/LKB1wt, S2: KRASmut/LKB1wt) H1299 isogenic clones. Six hours after CB-839 treatment, all clones showed 13C_5_-Gln intracellular accumulation, with a significant lowering of M + 5 glutamate (Glu) and no alteration in the extracellular Glu levels, indicating effective inhibition of glutamine catabolism exerted by CB-839 in all cells (Figure 2B and Supplementary Figure S3A). The decreased intracellular Glu was linked to the significant reduction of all isotopolugue fractions (M + 2, M + 3, M + 4, M + 5) of the TCA intermediates (fumarate, malate, citrate, succinate) in all clones, corroborating the evidence that glutamine metabolism maintains the TCA cycling (Figure 2C) [4]. There was also a significant overall drop in both unlabeled (M + 0) and labeled (M + 2, M + 3, M + 4) aspartate synthesis in all clones, further indicating that oxidative metabolism of glutamine was the main source for both TCA intermediates and intracellular aspartate formation (Figure 2C). The resistant clones showed a slight but significant rise in unlabeled aspartate levels compared to the sensitive ones, which did not come from the uptake of environmental aspartate (Supplementary Figure S3B) but might have come from alternative metabolic sources able to fuel the TCA cycle. Since CB-839 caused a comparable decrease in the TCA cycle activity in all clones, resistant cells might enhance lactate production through lactate dehydrogenase to maintain the cell redox state, which may be lowered with mitochondrial downregulation (Figure 2D). However, after CB-839 treatment, intracellular lactate levels did not change in resistant cells, indicating that these cells were not relying on increased lactate production to overcome the antiproliferative effect of CB-893 (Figure 2E). 

Interestingly, after CB-839 treatment, there was a significant drop in the intracellular alanine, coupled with a significant fall in extracellular alanine only in the resistant clones (Figure 2F,G). Therefore, we hypothesized that resistant clones rely on alanine catabolism to overcome the CB-839 glutamine impairment. Linked to the pyruvate metabolism is alanine aminotransferase (GPT2/ALT2), which catalyzes the reversible reaction generating pyruvate from alanine. Pyruvate-derived alanine could then be used to fuel mitochondrial metabolism in the presence of a scarcity of glutamine-derived alpha ketoglutarate and cellular biosynthesis through the generation of acetyl coenzyme A (acetyl-CoA).

We sought evidence that anabolic processes in the resistant cells were maintained by examining the intracellular levels of FFAs (palmitic, stearic and oleic acid synthesis) (Figure 2H). There were significant decreases in all the FFA levels in the sensitive clones, but not in the resistant ones after CB-839 (Figure 2I). These results are consistent with the ability of resistant clones to sustain cellular anabolic processes after glutamine withdrawal induced by CB-839 through the uptake and catabolism of alanine, in accordance with unlabeled aspartate formation. The reductions of all FFA levels in the sensitive clones indicated the activation of a catabolic process to feed the sensitive cell’s metabolism (Figure 2I).

### 3.3. Extracellular Alanine Catabolism Is Critical in Overcoming CB-839’s Antiproliferative Effect

The role of environmental alanine to supply cell metabolism is still only partially understood. Pancreatic cancer cells rely on extracellular alanine as a source of carbon to fuel the TCA cycle in a GPT2-dependent manner [18]. A recent publication reconstructed the role of extracellular alanine in T cell activation [19]. 

**Figure 3:**
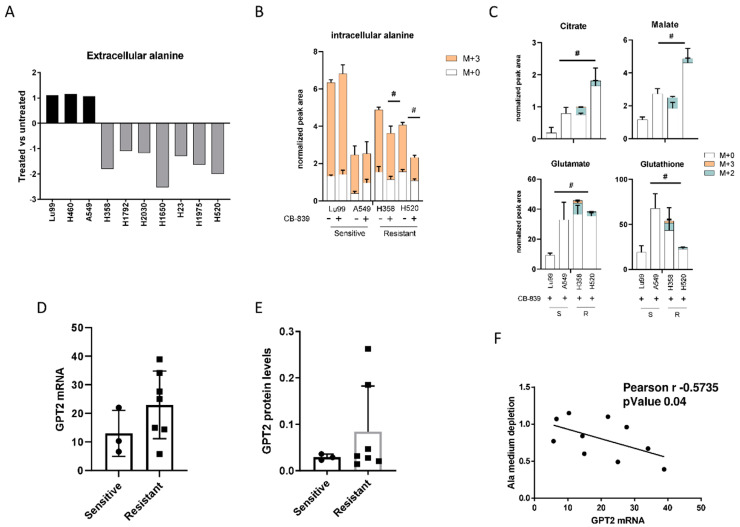
(**A**) Changes in extracellular alanine levels in the panel of ten NSCLC cell lines, CB-839-treated vs. untreated, cells (500 nM, 24 h treatment). (**B**) Unlabeled and 13C_3_-labelled alanine incorporation (M + 0, M + 3) in two sensitive (LU99, A549) and two resistant (H358, H520) cells, either untreated or CB-839 treated (500 nM, 24 h treatment). (**C**) The 13C_3_-alanine-derived carbon labelling (M + 2, M + 3, normalized peak area) of TCA cycle intermediates and citrate, malate, glutamate and glutathione derived metabolites in CB-839 treated sensitive and resistant cells. (**D**) GPT2 mRNA expression as RNA-seq, retrieved from the CCLE in the sensitive and resistant cells. (**E**) Western blot analyses of GPT2 expression in the NSCLC cell line panel. Ran was used as loading control. (**F**) Pearson correlation between alanine uptake from each cell line’s medium and GPT2 mRNA expression, retrieved from the CCLE. The average of three independent experiments is reported. Statistical significance ^#^ *p* < 0.05 refer to significant differences (one-way ANOVA and Tukey Kramer test) of M + 2 and M + 3 in the resistant vs. sensitive cells.

To demonstrate the role of alanine catabolism in overcoming the glutamine deprivation induced by CB-839, we profiled the extracellular alanine levels in all resistant and sensitive NSCLC cell lines previously tested for CB-839 activity. After CB-839 treatment, all the resistant cell lines had a significant depletion of the alanine levels in the cell medium, whereas in the sensitive lines, alanine levels were unchanged in treated and untreated cells (Figure 3A). Corroborating the role of alanine catabolism in CB-839 activity, we traced 13C_3_-alanine flux in two sensitive and two resistant NSCLC cells. Resistant (H358, H520) and sensitive (LU99, A549) cells did not show major differences in 13C_3_-alanine uptake capability, but resistant cells under CB-839 treatment showed lower intracellular M + 3 alanine levels (Figure 3B). Lower M + 3 alanine levels in the resistant cells were associated with the presence of 13C_3_-alanine-derived isotopolugues fraction (M + 2, M + 3) in both the former and later TCA cycle metabolites (citrate, malate) and TCA cycle derived glutamate and glutathione relative to the sensitive ones (Figure 3C). The role of 13C_3_-alanine catabolism in overcoming GLS1 inhibition was also confirmed in the resistant isogenic clones. Both R1 and R2 resistant cells treated with CB-839 showed M + 3 alanine incorporation and exploitation to generate TCA cycle intermediates (citrate, malate) and metabolic derivates (glutamate, glutathione and aspartate) (Supplementary Figure S4A,B). Being alanine aminotransferase 2 (GPT2), the enzyme responsible of alanine catabolism [20,21], we analyzed both GPT2 mRNA (Figure 3D) and protein levels (Figure 3E), observing an increase in the resistant cell lines compared to the sensitive counterpart, even though it was not statistically significant.

**Figure 4:**
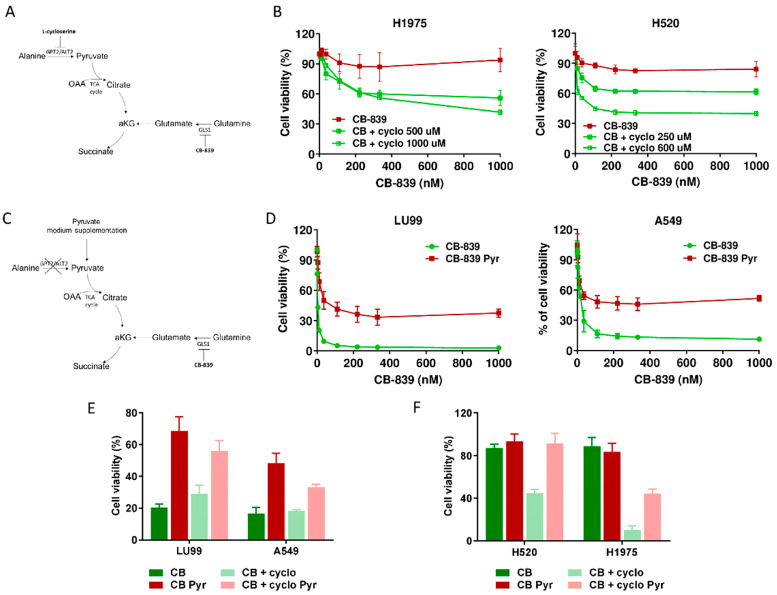
(**A**) Schematic representation of metabolic strategy to overcome CB-839 resistance, targeting GPT2 by l-cycloserine (cyclo). (**B**) Dose–response curves of H1975 (left panel) and H520 (right panel) cells treated with increasing concentrations of CB-839, alone (CB-839) or with l-cycloserine (CB + cyclo) at the reported concentrations. The response to the drugs was assessed with the MTS assay 72 h from the treatment start. (**C**) Schematic representation of metabolic strategy to induce CB-839 resistance in the sensitive cell lines by pyruvate supplementation, with inhibition of GLS1, alone or together with GPT2. (**D**) Dose–response curves of LU99 (left panel) and A549 (right panel) cells treated with increasing concentrations of CB-839, grown and treated in standard (CB-839) or pyruvate-enriched medium (CB-839 Pyr). The response to the drug was assessed with the MTS assay 72 h from treatment start. (**E**) Histograms of LU99 and A549 cells grown in standard or pyruvate-enriched medium (Pyr) and treated with CB-839 (CB, 12 nM for LU99 and 111 nM for A549) and l-cycloserine (cyclo, 75 µM for LU99 and 100 µM for A549), either alone or in combination. The response to the drug was assessed 72 h from the start of treatment with the MTS assay. (**F**) Histograms of H520 and H1975 cells grown in standard or pyruvate-enriched medium (Pyr) and treated with CB-839 (CB, 1000 nM) and l-cycloserine (cyclo), 250 µM for H520 and 500 µM for H1975), either alone or in combination. The response to the drug was assessed with the MTS assay 72 h from treatment start. The average of three independent experiments is reported. For the sake of clarity, the statistical significance one-way ANOVA and Bonferroni post-test for multiple comparisons (panel **B**,**D**,**E**,**F**) are reported as Supplemental Table S1, Table S2, Table S3 and Table S4, respectively).

We also observed a high, inverse, statistically significant correlation between the extent of extracellular alanine depletion in the resistant cells and their GPT2 mRNA expression (Figure 3F). Because of the role of GPT2 in converting alanine into pyruvate, we concluded that the GPT2-mediated pyruvate supply was responsible for growth and survival during GLS1 inhibition in resistant NSCLC cells. Pyruvate is the principal substrate for mitochondrial metabolism and has been reported to compensate glutamine depletion by refueling the TCA cycle and sustaining cancer anabolic processes by exploiting the activity of different enzymes such as the pyruvate dehydrogenase complex or pyruvate carboxylase [22,23,24]. To demonstrate this, we treated the CB-839-resistant cell lines H1975 and H520 with sub-toxic concentrations of the GPT2 inhibitor l-cycloserine (Supplementary Figure S5) in combination with increasing concentrations of CB-839 (Figure 4A). The pharmacological inhibition of GPT2 sensitized H1975 and H520 cells to the GLS1 inhibitor, indicating that GPT2-mediated pyruvate production is instrumental for cell growth during CB-839 treatment (Figure 4B and Supplementary Table S1).

To further confirming these results, we supplemented the medium of two CB839-sensistive NSCLC cell lines with exogenous pyruvate and let the cells grow for about one week before CB-839 treatment (Figure 4C). When cells were exposed to the GLS1 inhibitor, pyruvate supplementation abrogated CB-839 activity in both LU99 and A549 sensitive cells, strongly supporting the key role of GPT2-mediated pyruvate supply in driving cell survival (Figure 4D and Supplementary Table S2). Finally, we verified whether GTP2 pharmacological inhibition rescued the sensitivity when pyruvate was exogenously supplied to both CB-839-responsive and non-responsive cells. When l-cycloserine was administered in combination with CB-839 to LU99 and A549 (sensitive), H520 and H1975 (resistant) cells grown in pyruvate-enriched medium, there was a complete lack of sensitivity to the treatment (Figure 4E,F and Supplementary Tables S3 and S4). 

### 3.4. Expression of GPT2 in Target Cancer Patients: Implications for CB-839 Response

**Figure 5:**
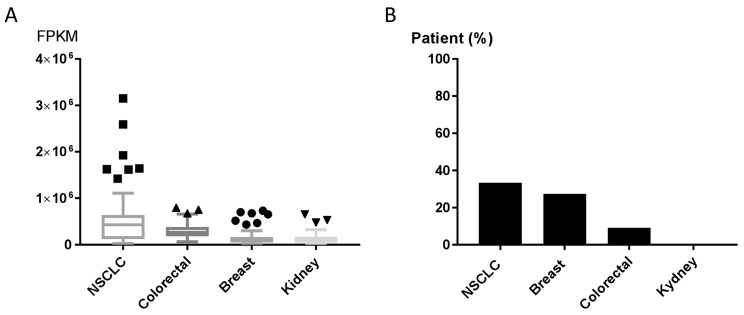
(**A**) GPT2 RNA expression as RNA-seq data retrieved from The Cancer Genome Atlas (TCGA) for NSCLC, colorectal, breast and kidney cancer patients. (**B**) GPT2 protein levels; bars indicate the percentage of patients (12 patients) with high and medium protein expression levels obtained from the Human Protein Atlas [25].

The GLS1 inhibitor CB-839 is currently undergoing early-phase clinical trials in different human tumors, including NSCLC (NCT02771626, NCT03831932), triple-negative breast cancer (NCT03875313), renal cell carcinoma (NCT03163667) and metastatic colon cancer (NCT03263429, NCT03875313, NCT02771626). Results about patients’ responses are not available yet. We explored the expression of GPT2 in human tumor using the Cancer Genome Atlas (TCGA) and the Human Protein Atlas [25] to assess the GPT2 mRNA and protein levels in tumor patients potentially eligible for CB-839 treatment (clinicaltrials.gov [26]. The expression levels of the GPT2 gene varied in NSCLC, breast, colorectal and kidney cancer tissues (70 specimens/tumor). NSCLC samples had high GPT2 expression and dispersion, whereas kidney cancers had lower and more homogeneous expression (Figure 5A). These different trajectories were confirmed by the immunohistochemistry (IHC) analyses in which 33% of NSCLC and 27% of breast cancer tissues had high/medium GPT2 protein levels, respectively, while kidney cancer had no detectable GPT2 protein (Figure 5B).

In conclusion, we identified the role of alanine-derived pyruvate catabolism in driving NSCLC cell survival after glutaminase inhibition. As alanine is the second most abundant amino acid in circulation after glutamine, reaching plasma micro molar concentrations, it may be highly accessible for cancer cells and thus act as an important metabolic mechanism for overcoming treatment. 

Our results indicated that the response to the glutaminase inhibitor CB-839 in NSCLC cell lines was not linked to specific genetic alterations, but rather depended on the individual cell’s ability to rely on GPT2 to generate pyruvate from alanine and compensate the reduced glutamate availability. Inhibition of GPT2 by l-cycloserine was in fact effective in rescuing cell sensitivity to glutaminase inhibition.

Our findings suggest that it could be useful to expand the measurement of GPT2 protein levels to stratify NSCLC patients and select the potential best responders. Finally, as l-cycloserine is an antibiotic used for tuberculosis, its safety and tolerability profiles [27] have already been confirmed in specific clinical trials and drug surveillance studies. Therefore, repurposing l-cycloserine for patients with poor chances of benefit from CB-839 single treatment might be successful to increase the tumor response with rapid clinical impacts at lower costs than de novo drug development.

## Figures and Tables

**Table 1:** *KRAS*, *LKB1* and *KEAP1* mutational status and *KRAS* copy number variation (CNV) of the NSCLC cells used, obtained by the COSMIC database [16]. Overexp: overexpression without a clear gene duplication, red: CB-839 resistant cell lines, green: CB-839 sensitive cell lines.

CELL LINE	KRAS	KRAS CNV	LKB1	KEAP1
H1792	G12C	NO	WT	LOSS
H1975	WT	-	WT	WT
H358	G12C	OVEREXP	WT	WT
H2030	G12C	NO	LOSS	LOSS
H520	WT	-	WT	WT
H358_7	G12C	-	LOSS	WT
H23	G12C	OVEREXP	LOSS	LOSS
H1650	WT	-	WT	WT
H460	Q61H	NO	LOSS	LOSS
A549	G12S	NO	LOSS	LOSS
LU99	G12C	5	WT	WT
LU99_2	G12C	-	LOSS	WT

## Supplementary Materials

The following are available online at https://www.mdpi.com/2073-4409/9/8/1766/s1, Figure S1: A. Dose–response curves of 6 NSCLC cell lines treated with increasing concentrations of CB-839 using SRB assay. B. DNA content, obtained by flow cytometric analysis, after 24 h or 48 h of treatment with CB-839 in sensitive and resistant cell lines; Figure S2: Dose–response curves of sensitive and resistant NSCLC H1299 isogenic cell clones treated with increasing concentrations of CB-839; Figure S3: Extracellular aspartate levels with or without CB-839 in two sensitive and two resistant H1299-derived clones; Figure S4: A. The 13C-labelled alanine incorporation in the resistant NSCLC H1299 isogenic cell clones treated with CB-839. B. The 13C-alanine-derived carbon labelling of TCA cycle intermediates and derived metabolites in CB-839 treated resistant H1299-derived clones; Figure S5: Dose–response curves of H1975 and H520 cells treated with increasing concentrations of l-cycloserine; Table S1: Significant differences of the data presented in Figure 4B; Table S2: Significant differences of the data presented in Figure 4D; Table S3: Significant differences of the data presented in Figure 4E; Table S4: Significant differences of the data presented in Figure 4F.
