# Supplementary material for: Glutaminase Inhibition on NSCLC Depends on Extracellular Alanine Exploitation
Source: Cells. 2020 Jul 23;9(8):1766. doi: 10.3390/cells9081766 (PMC7465377; doi:10.3390/cells9081766)
Supplement: Supplementary file 1 [file cells-09-01766-s001.pdf]

A

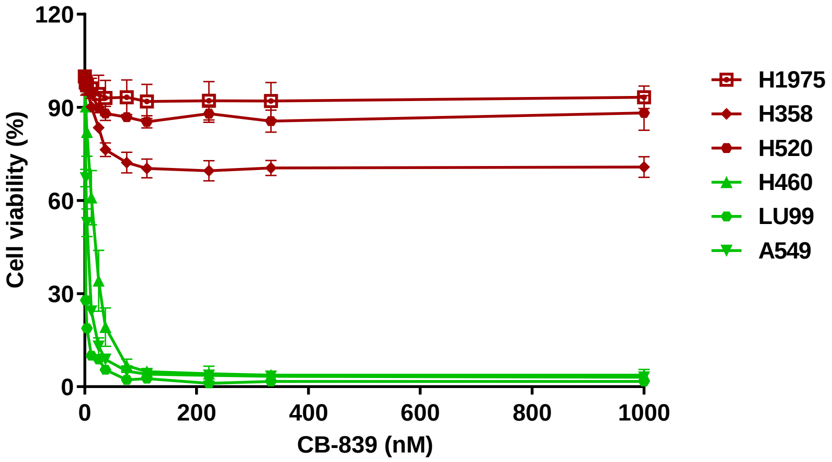

B

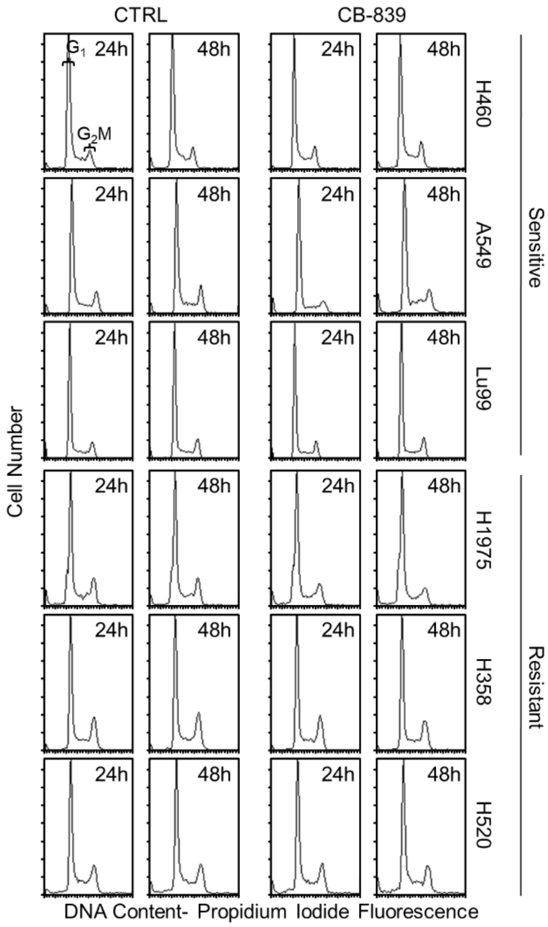

Figure S1. A Dose-response curve of three sensitive (H460, LU99, A549) and three resistant (H1975, H358, H520) NSCLC cell lines treated with increasing concentrations of CB-839. The response to the drug was evaluated 72h from the start of treatment with the Sulforhodamine B assay. The average of three independent experiments is reported. B. DNA histograms of three sensitive (H460, LU99, A549) and three resistant (H1975, H358, H520) NSCLC cell lines untreated or treated with 500 nM CB-839 for 24h and 48h. The analysis was performed through flow cytometry.

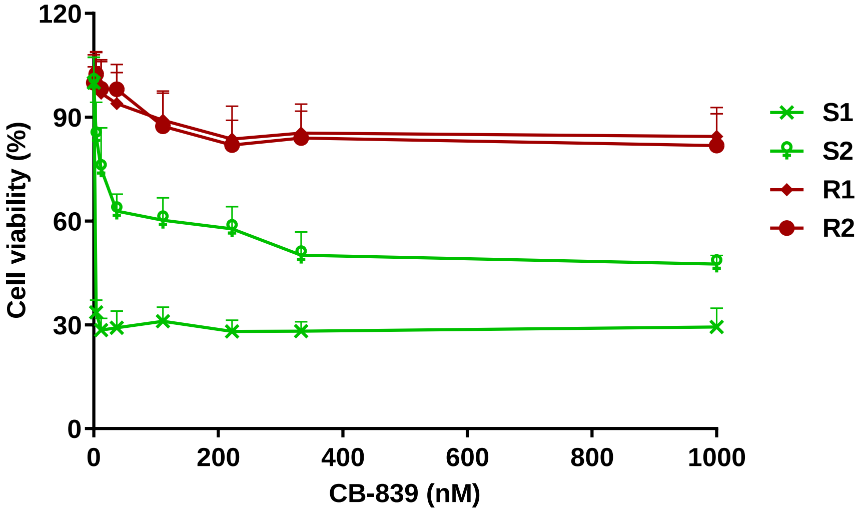

Figure S2. Dose-response curve of sensitive (S1 KRASwt/LKB1wt, S2 KRASmut/LKB1wt) and resistant (R1 KRASwt/LKB1del, R2 KRASmut/LKB1del) NSCLC H1299 isogenic cell clones treated with increasing concentrations of CB-839. The response to the drug was evaluated 72h from the start of treatment with the MTS assay. The average of three independent experiments is reported. Statistical significance: * $p<0.05$ ** $p<0.01$ *** $p<0.001$ **** $p<0.0001$, computed by two-way ANOVA and Bonferroni post-test for multiple comparisons is reported in the table below for clarity sake.

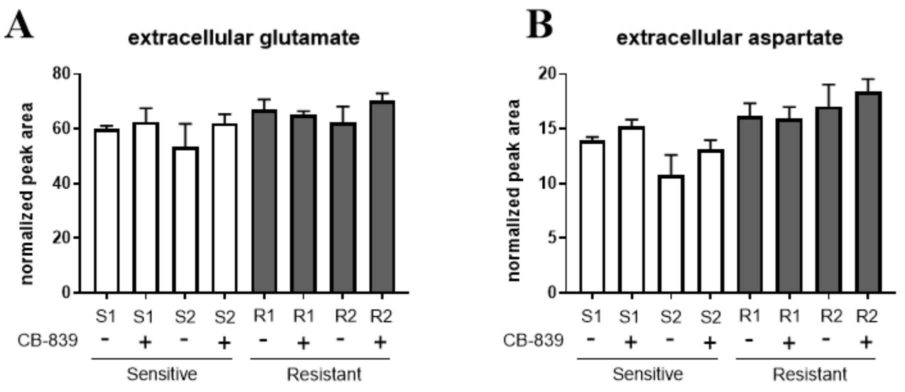

Figure S3. Extracellular Glutamate (A) and Aspartate (B) levels (normalised peak area) with or without CB-839 (500 nM, 24h of treatment) in two sensitive (S1 KRASwt/LKB1wt, S2 KRASmut/LKB1wt) and two resistant (R1 KRASwt/LKB1del, R2 and KRASmut/LKB1del) H1299-derived clones. Average of three independent experiments is reported.

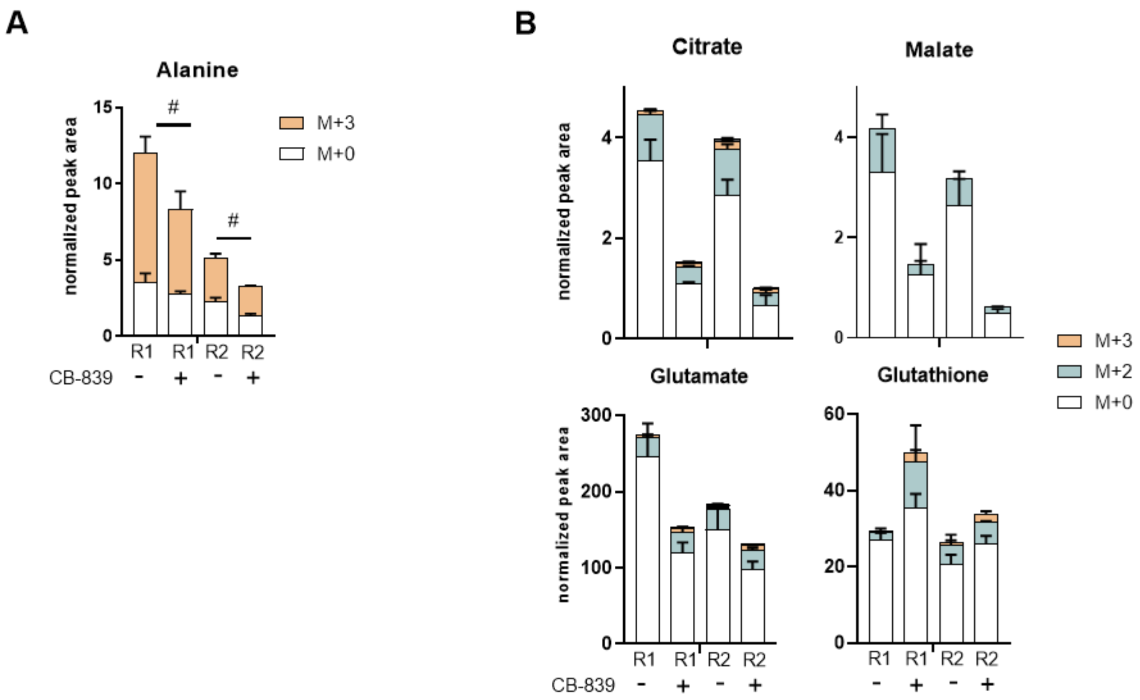

Figure S4. A. Unlabelled and 13C-labelled alanine incorporation (M+0, M+3, normalised peak area) with or without CB-839 (500 nM, 24h of treatment) in two resistant (R1 KRASwt/LKB1del, R2 and KRASmut/LKB1del) H1299-derived clones. **B.** 13C-alanine-derived carbon labelling (M+2, M+3, normalised peak area) of TCA cycle intermediates and derived metabolites citrate, malate, glutamate and glutathione in CB-839 treated resistant (R1 KRASwt/LKB1del, R2 and KRASmut/LKB1del) H1299-derived clones. Average of three independent experiments is reported. Statistical significance # p<0.05 refer to significant differences (one-way ANOVA and Tukey Kramer test) of M+3 in the CB-839 treated vs untreated cells.

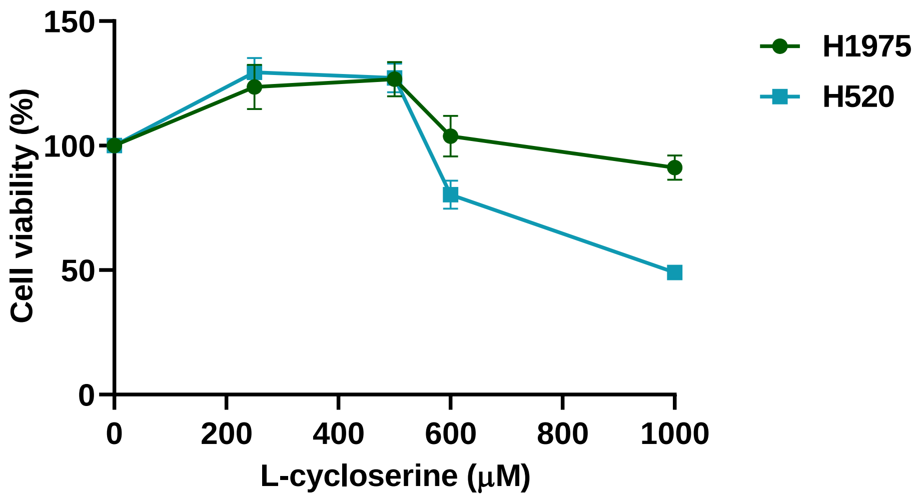

Figure S5. Dose-response curves of H1975 and H520 cells treated with increasing concentrations of L-cycloserine. The response to the drug was evaluated 72h from the start of treatment with the MTS assay. The average of three independent experiments is reported.

Table S1. Significant differences of the data presented in figure 4B. cyclo: L-cycloserine; CB: CB-839. Two-way ANOVA and Bonferroni multiple comparison post-test. * p<0.05 ** p<0.01 *** p<0.001 **** p<0.0001.

.	CB-839 (nM)						
	**4**	**12**	**37**	**111**	**222**	333	1000
**S1 vs. S2**	****	****	****	****	****	**	*
**S1 vs. R1**	****	****	****	****	****	****	****
**S2 vs. R2**	*	**	****	***	***	****	****
**R1 vs. R2**	ns	ns	ns	ns	ns	ns	ns

Table S2. Significant differences of the data presented in figure 4D. Pyr: pyruvate supplemented RPMI medium. Two-way ANOVA and Bonferroni multiple comparison post-test. **** p<0.0001.

	**LU99 - CB-839 (nM)**							
	**1**	**4**	**12**	**37**	**111**	**222**	**333**	**1000**
**CB-839 vs. CB-839 Pyr**	****	****	****	****	****	****	****	****
	**A549 - CB-839 (nM)**							
	1	4	12	37	111	222	333	1000
**CB-839 vs. CB-839 Pyr**	ns	ns	ns	ns	****	****	****	****

Table S3. Significant differences of the data presented in figure 4E. CB: CB-839, cyclo: L-cycloserine, Pyr: pyruvate supplemented RPMI medium. One-way ANOVA and Bonferroni multiple comparison post-test. * p<0.05 *** p<0.001 **** p<0.0001.

	**H1975 - CB-839 (nM)**						
	**4**	**12**	**37**	**111**	**222**	**333**	**1000**
**CB-839 vs. CB + cyclo 500 µM**	ns	ns	*	*	***	***	****
**CB-839 vs. CB + cyclo 1000 µM**	ns	ns	ns	*	**	***	****
**CB + cyclo 500 ⚜M vs. CB + cyclo 1000 µM**	ns	ns	ns	ns	ns	ns	ns
	**H520 - CB-839 (nM)**						
	12	37	111	222	333		1000
**CB-839 vs. CB + cyclo 500 µM**	**	***	****	****	****		****
**CB-839 vs. CB + cyclo 1000 µM**	****	****	****	****	****		****
**CB + cyclo 500 ⚜M vs. CB + cyclo 1000 µM**	****	****	****	****	****		****

Table S4. Significant differences of the data presented in figure 4F. CB: CB-839, cyclo: L-cycloserine, Pyr: pyruvate supplemented RPMI medium. One-way ANOVA and Bonferroni multiple comparison post-test. ** p<0.01 *** p<0.001 **** p<0.0001.

	**H520**	**H1975**
**CB vs. CB Pyr**	ns	ns
**CB vs. CB + cyclo**	***	****
**CB Pyr vs. CB + cyclo Pyr**	ns	***
**CB + cyclo vs. CB + cyclo Pyr**	****	**
